# Learning Diatoms Classification from a Dry Test Slide by Holographic Microscopy

**DOI:** 10.3390/s20216353

**Published:** 2020-11-07

**Authors:** Pasquale Memmolo, Pierluigi Carcagnì, Vittorio Bianco, Francesco Merola, Andouglas Goncalves da Silva Junior, Luis Marcos Garcia Goncalves, Pietro Ferraro, Cosimo Distante

**Affiliations:** 1 Institute of Applied Sciences and Intelligent Systems (ISASI) National Research Council (CNR) of Italy, Via Campi Flegrei 34, 80078 Pozzuoli, NA, Italy; p.memmolo@isasi.cnr.it (P.M.); f.merola@isasi.cnr.it (F.M.); p.ferraro@isasi.cnr.it (P.F.); 2 Institute of Applied Sciences and Intelligent Systems (ISASI) National Research Council (CNR) of Italy, Via Monteorni snc University Campus, 73100 Lecce, Italy; p.carcagni@isasi.cnr.it (P.C.); c.distante@isasi.cnr.it (C.D.); 3 Department of Computer Engineering and Automation, Federal University of Rio Grande do Norte, 59078 Natal, Brazil; andouglasjr@gmail.com (A.G.d.S.J.); lmarcos@dca.ufrn.br (L.M.G.G.)

**Keywords:** environmental monitoring, digital holography, deep learning, diatoms, marine pollution, water quality sensors, classification, microplankton, taxonomy, phase-contrast microscopy

## Abstract

Diatoms are among the dominant phytoplankters in marine and freshwater habitats, and important biomarkers of water quality, making their identification and classification one of the current challenges for environmental monitoring. To date, taxonomy of the species populating a water column is still conducted by marine biologists on the basis of their own experience. On the other hand, deep learning is recognized as the elective technique for solving image classification problems. However, a large amount of training data is usually needed, thus requiring the synthetic enlargement of the dataset through data augmentation. In the case of microalgae, the large variety of species that populate the marine environments makes it arduous to perform an exhaustive training that considers all the possible classes. However, commercial test slides containing one diatom element per class fixed in between two glasses are available on the market. These are usually prepared by expert diatomists for taxonomy purposes, thus constituting libraries of the populations that can be found in oceans. Here we show that such test slides are very useful for training accurate deep Convolutional Neural Networks (CNNs). We demonstrate the successful classification of diatoms based on a proper CNNs ensemble and a fully augmented dataset, i.e., creation starting from one single image per class available from a commercial glass slide containing 50 fixed species in a dry setting. This approach avoids the time-consuming steps of water sampling and labeling by skilled marine biologists. To accomplish this goal, we exploit the holographic imaging modality, which permits the accessing of a quantitative phase-contrast maps and a posteriori flexible refocusing due to its intrinsic 3D imaging capability. The network model is then validated by using holographic recordings of live diatoms imaged in water samples i.e., in their natural wet environmental condition.

## 1. Introduction

Water quality assessment is the overall process of evaluation of the physical, chemical and biological nature of water and, nowadays, it is one of the most challenging tasks tackled by research scientists worldwide [1,2]. Among all monitoring techniques, microscopy imaging of microorganisms in marine and freshwater habitats plays a crucial role since the dominant phytoplankters, namely diatoms, are unicellular biomarkers of seawater quality [3,4]. In fact, diatoms are very sensitive to changes in environmental conditions that might occur due to the presence of pollutants and are not observable in other planktons. This is due to their inner fine structures, namely chloroplast, whose shapes’ variations are linkable to the presence of contaminants. Moreover, the diversity of chloroplast shapes, their number and location within cells, are highly distinctive features for diatoms taxonomy [5]. Due to the huge number of species, the classification problem is very challenging, making necessary the use of advanced technology and expert staff, although methods for automatic identification and classification, based on classical pattern recognition and computer vision techniques, have been proposed to overcome this limitation [6,7]. In particular, The Automatic Diatom Identification and Classification (ADIAC) project is a reference in the investigation of diatoms analysis systems [6] and provides a dataset of about 10,000 diatoms images, mostly captured directly from the microscope. Other populations were photographed using monochrome film and the developed negative was acquired through a slide scanner.

Recently, the use of artificial intelligence in microscopy has deeply marked the field of biological samples investigation, paving the way to remarkable advances in imaging and automatic classification [8,9,10,11,12,13,14,15]. Among them, label-free quantitative imaging of cells employed in learning-based classification is one of the most popular research topics nowadays [16,17]. Differently from the classical machine learning approach that employs distinctive image features identified ad hoc to address a specific classification problem, the deep learning strategy uses multilayered Convolutional Neural Networks (CNNs) for blind and automated image analysis, thus optimizing the image features selection [9]. Learning approaches were successfully applied in challenging problems in microscopy imaging, ranging from human diseases identification [18,19,20,21] to the marine micro-organisms’ classification, such as diatoms taxonomy [22,23,24,25,26]. In particular, in [22] the Legendre polynomial shape descriptors and principal component analysis were successfully combined for the *Cymbella cistula* species identification, by using conventional microscope imaging. A hierarchical multi-label classification system was demonstrated in [23] for diatom image classification using the ADIAC database [6]. This approach, based on ensembles of predictive clustering trees, was able to simultaneously predict all different levels in the hierarchy of taxonomic ranks, such as genus, species, variety, and form of diatoms. The best results for ADIAC dataset, with up to 97.97% accuracy, have been obtained with 38 classes using Fourier and SIFT descriptors with a random forest classifier. In [24], the hand-crafted method has been proposed, where a set of fixed features was selected by expert knowledge. However, these methods present limited results as in [25], where 14 classes were classified with Support Vector Machine (SVM) reaching a final accuracy of 94.7%. Approaches based on CNNs have been proposed in [26], where an extensive dataset was specifically collected (80 classes with 100 samples/class, bright-field images), covering different illumination conditions, and it was computationally augmented to more than 160,000 samples. They employed the AlexNet model [9] reaching an overall accuracy of 99%. Another work used approximately 100,000 images from nine phytoplankton populations [27], employing one pre-trained Resnet50 network and achieving nearly 97% accuracy. To the best of our knowledge, all learning-based approaches reported in the literature relied on bright-field and/or epifluorescence images of diatoms for classification purposes. Alternative imaging modalities such as Digital Holography (DH) in transmission microscopy configuration have been recently demonstrated to be very effective in detecting diatom structures [28,29,30,31], thanks to the flexible digital refocusing by post-processing back-propagation and phase-contrast imaging capabilities. Moreover, the use of DH imaging in combination with classical machine learning has been recently demonstrated for the identification of micro-plastics, discerning them from diatoms [32,33]. DH provided the possibility to measure unique image features related to the quantitative phase information that are highly distinctive in classifying micro-plastics from diatoms. In particular, 10,000 samples belonging to a heterogeneous micro-plastic class and nine diatoms populations were classified by the SVM reaching a classification accuracy higher than 99% [32]. However, also in this study, skilled personnel had to create the dataset with labeled and separated diatom species before the holographic data capture. To the best of our knowledge, any previous study concerning diatom species classification required expert diatomists for the dataset labelling and a large amount of training data for each class. Due to the large heterogeneity of the microalgae population, performing an exhaustive training of a network called to discriminate between the thousands different species is an unfeasible task. However, test slides containing various sets of diatoms species are becoming more and more accessible on the market [34]. These are usually prepared by expert marine biologists for taxonomy purposes, by placing the elements of each species side by side in between two glass slides. Live diatoms are used for this scope, while making sure to preserve their morphology and chloroplast content. If a proper selection of the species is made, such glass slides can be thought of as libraries of the populations that can be found in a certain portion of marine water and could become in the next future a sort of fingerprint of the marine habitat.

In order to tackle the problem of finding a large enough dataset to classify diatoms by deep learning approaches, here we exploit one such test slide to generate images for training the network. For each class, one single holographic image is captured in transmission configuration, then this is used to generate a fully augmented dataset of phase images of diatoms belonging to the same population through proper transformations applied to the phase-contrast map. The augmented datasets of all the classes contained in the test slide are used to train a deep learning architecture. In particular, we used a commercial glass slide containing 50 species. Notably, we trained the network by imaging the test slide containing diatoms in a dry condition, thus greatly simplifying the recording stage. We demonstrate that networks trained in this way are able to accurately classify morphologically similar species of live diatoms even when these are imaged in a Petri dish in a liquid environment through a digital holographic microscope. We investigated and tested thirteen network models and we ranked them on the basis of the classification accuracy they achieved in the test stage. Then, we selected a proper network subset and considered their ensemble model. In this approach, the output of each trained model is combined with the aim to obtain the final accuracy greater than each individual model. There are several techniques that could be used to build an ensemble model. More basic ones are max voting, averaging, and weighted average. More complex methods are Stacking, Blending, Bagging, and Boosting. In this work, we used the max voting approach where the most voted result is chosen as the final class. We show that the ensemble outperforms each single model and reaches a 98% classification accuracy. This proves the powerful capability of deep learning methodologies to generalize well when trained with fully simulated images. Furthermore, the network models turn out to be robust against changes of the imaging condition, e.g., dry or wet environment in our case. At this scope, the large homogeneity of live diatoms samples within the same species helps to relax the problem complexity and promotes an accurate classification through pre-trained architectures. In the next future, a further refinement of the selection of the diatoms filling the test slide could be used to create datasets well-tailored to more and more specific marine habitats. It is worth mentioning that holographic microscopes have become over the years more and more compact, light in weight and cost-effective [35,36,37,38]. Thus, holographic imaging coupled to deep learning can be used out of the lab for environmental monitoring during field sampling campaigns, and in the form of a widespread sensor network allocated on autonomous systems.

## 2. Materials and Methods

### 2.1. Holographic Acquisition: Training Based on a Commercial Glass Slide

**Figure 1:**
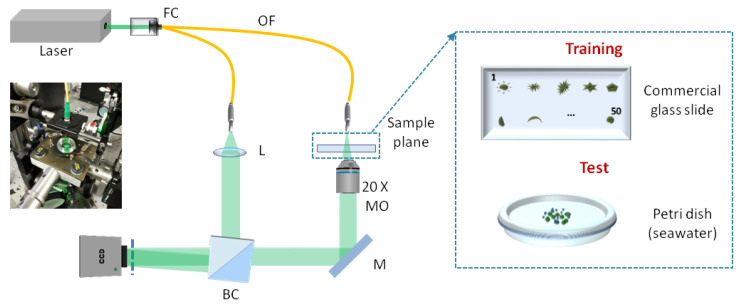
Experimental setup. FC: Fiber coupler; OF: Optical Fiber; BC: Beam Combiner; M: Mirror; MO: Microscope Objective; L: lens.

The experimental setup is a Mach-Zehnder interferometer in transmission configuration. High coherence light is emitted by a solid-state laser working at λ = 532 nm wavelength and 400 mW power at the laser exit. The laser is fiber-coupled, and optical fibers are used to split light into a reference beam and an object beam. The reference beam directly reaches the sensor with an off-axis illumination angle. The object beam goes through the sample, is collected by a 20× microscope objective and then reaches the Charge Coupled Device (CCD), where it interferes with the reference beam and creates a pattern of interference fringes, i.e., the hologram. The CCD has 2048 × 2048 pixels with 5.5 μm pixel size. Figure 1 sketches the experimental setup and shows a photograph of the sample plane captured during the test stage. As shown in Figure 1, during the experiment devoted to acquiring images to be used for training, a commercial glass slide was used in the sample plane. The test target was 2 mm thick, and made of two glass slides containing in between them 50 different species of fixed diatoms. In the target under test, diatoms span over a large Field of View (FoV), with specific diameters going from 50 µm to 200 µm. In order to collect the entire set of diatoms, we captured 7 holograms after scanning the glass slide using a motorized linear stage. The holograms of the glass slide have been used to create the dataset to train the network, as discussed in the following sections. During the test stage, the glass slide was replaced with a Petri dish containing diatoms dipped in seawater (Figure 1). Diatoms cultures were diluted with sterile seawater and maintained at room temperature until the experiments were performed. In this case, each object was acquired out of focus, and then numerically refocused.

### 2.2. Hologram Reconstruction and Data Augmentation

Let H, O and *R* be the digital hologram, the object beam and the reference beam, respectively. The captured hologram is the intensity of the interference between *O* and *R*:

(1) $$H={\left|R+O\right|}^{2}={\left|R\right|}^{2}+{\left|O\right|}^{2}+2\left|R\right|\left|O\right|cos\left[2\pi f_{R}\left(x+y\right)-\varphi_{R}+\psi_{o}\right],$$

where we assumed *R* to be a tilted plane wave, with phase $\varphi_{R}$, forming an angle ϑ with the object beam, so that $O=\left|O\right|e^{j\psi_{o}}$ modulates in amplitude and phase a spatial carrier with frequency $f_{R}=(\sin\vartheta )/\lambda$. Hologram demodulation returns the complex object wave. Numerical propagation methods solve the diffraction integral under proper assumptions, and allows linking the object wavefront in the acquisition plane to the object wavefront that propagates in any plane along the optical axis. We used the Angular Spectrum method to solve the diffraction integral. The reconstructed complex amplitude of the object in the image plane is $C\left(x_{R},y_{R};\lambda ;z\right)={Ρ}_{z}\left\{H_{dem}\right\}$, where $H_{dem}$ is the demodulated hologram, $\left(x_{R},y_{R}\right)$ is the reconstruction plane at distance z from the hologram plane, and $P_{z}\left\{\ldots \right\}$ is the propagation operator. In principle, $P_{z}$ can be applied several times to $H_{dem}$ while varying the propagation distance z. Thus, from $H_{dem}$ a stack of reconstructed complex images of the object can be obtained, which are defocused in different ways and correlated through the object diffraction pattern. The object in focus and, in turn, its phase-contrast map, $\psi_{o,F}$, are obtainable by propagating the hologram to the best focus distance, $z=z_{F}$, which can be estimated by optimizing proper contrast metrics. In particular, the best focus distance is chosen by a commonly used automatic refocusing criterion, i.e., the minimization of the Tamura coefficient of the amplitude image, obtained at different propagation distances. The refocused phase-contrast map is then used as input for the testing.

### 2.3. The Dataset

**Figure 2:**
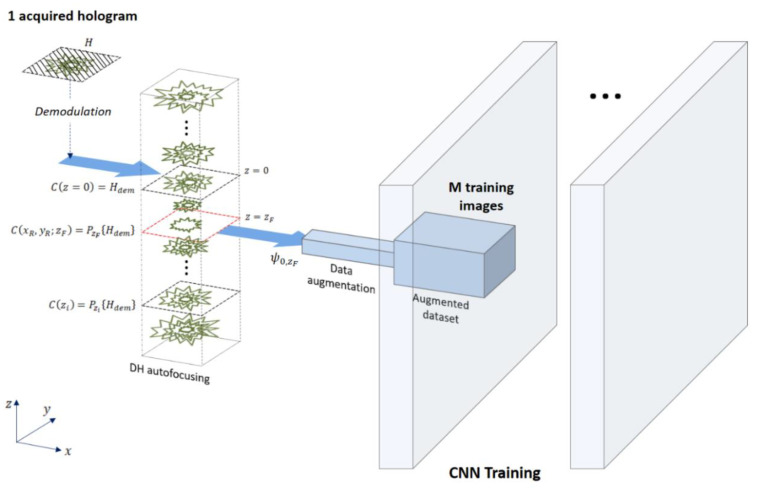
Augmentation of Holographic data provides 174.636 phase-contrast images from one single hologram of the object.

**Figure 3:**
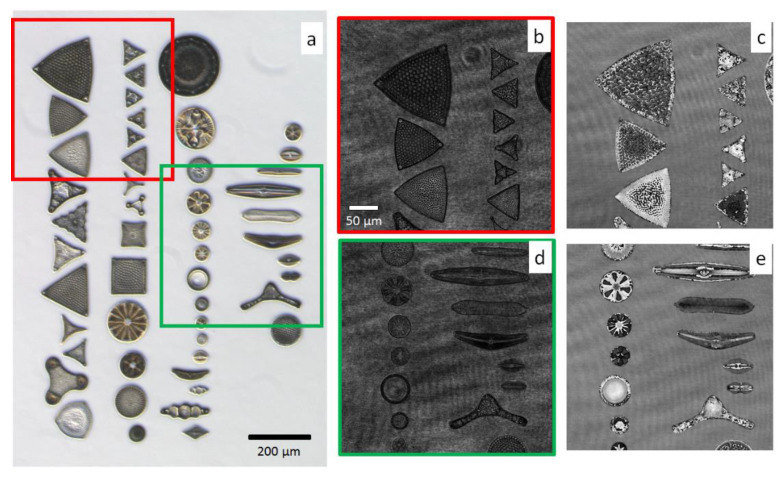
Holographic recording and reconstructions of diatoms within the glass slide. (**a**) Bright field image of all diatoms on the glass slide (5× commercial microscope). (**b**,**d**) are two recorded digital holograms within the red and green Field of View (FoV), respectively, and (**c**,**e**) are the corresponding wrapped quantitative phase images (WQPIs) reconstructions.

The main idea behind this work, represented in Figure 2, is to exploit the DH image of the test slide to create the dataset for training the CNN. In other words, a fully augmented dataset is created by data augmentation starting from one single recorded hologram per class. In particular, from the hologram of an object belonging to a certain class, we performed the process of in-focus holographic reconstruction, and thus a complex valued image was obtained from which the phase can be retrieved. Then, we applied conventionally used 2D data augmentation methods resulting in an augmentation of the number of simulated images potentially up to 174.636 per class, i.e., a total dataset size of 8.731.800 elements. This large dataset was obtained at minimum cost (one single captured hologram) and enabled one to feed a CNN in order to train it to classify different diatoms populations. In the case of marine microplankton, training the CNN with commercially available glass slides consisting of one sample per class and using it to recognize real samples in seawater is a very interesting opportunity to create a large database of marine microalgae species [34]. In our case, we obtained the initial measured images of the training dataset from the quantitative phase reconstructions of recorded holograms of the commercial glass slide. Since the imaging FoV was smaller than the size of the test slide containing the fifty species, seven holograms were acquired after scanning the glass slide using a motorized stage. Then the wrapped quantitative phase images (WQPIs) were calculated by holographic reconstruction, and each diatom was isolated by the image segmentation process, based on the Otsu method followed by image filling, thus generating a binary mask for each diatom. In Figure 3, a bright field image of the entire glass slide is reported (Figure 3a) along with two digital holograms recorded within the red and the green regions (Figure 3b,d). The corresponding WQPIs are recovered by applying the holographic reconstruction process (Figure 3c and Figure 3e, respectively).

After segmenting the holographic reconstructions, we obtain 50 wrapped WQPIs of single diatoms. Each WQPI is used as the generator for all images belonging to the class. In particular, we use 3 nested transformations, which are described in the following:

- 21 × 21 possible size scale in the range [−20%,+20%] × [−20%,+20%] around the initial QPI size by using the image resizing.
- 36 possible image orientation, by applying a 10 degree rotation step to the WQPI.
- 11 possible phase shift biases, taking in the uniformly distributed interval [0,π], that could be caused by random phase offsets during the recording and/or reconstruction processes (phase offsets as residual errors in the aberration compensation step).

It is important to note that each WQPI has gray levels varying in the range [−π,+π]. Typically, image transformation processes employ some interpolation algorithms that could push the image values out of such range. Therefore, after each transformation, the 2π-modulation operator is applied to the image to be sure that it maintains the WQPI format.

**Figure 4:**
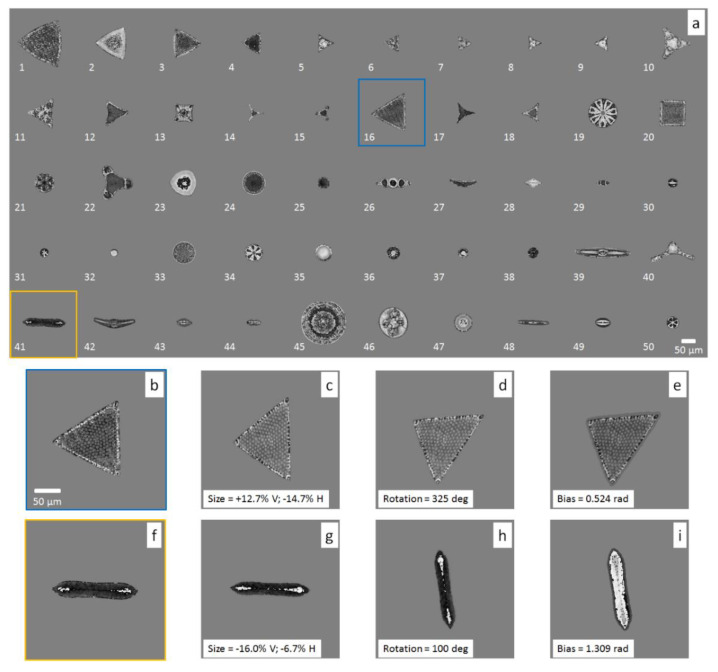
Initial guess for creating the training dataset. (**a**) WQPIs of each diatom in the test glass slide, labeled from 1 to 50. (**b**,**f**) are two WQPIs selected among the others, on which a cascade of transformations are applied, i.e., resizing (**c**,**g**), rotation (**d**,**h**) and phase biasing (**e**,**i**).

In Figure 4a, we report all the reconstructed WQPIs segmented from the reconstructions of the recorded holograms. We labeled the diatom classes with integers ranging from 1 to 50. Two examples of how the proposed data augmentation works are reported in Figure 4b–e and Figure 4f–i for diatoms 16 and 41, respectively. The transformation grafting created two new simulated WQPIs in Figure 4e,i.

**Figure 5:**
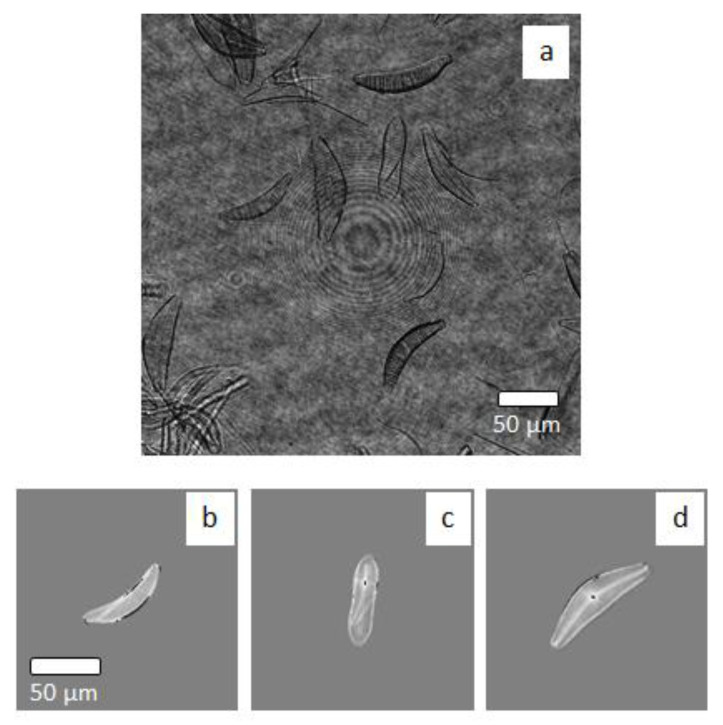
Examples of holographic images of live diatoms. (**a**) one of the recorded digital holograms of diatoms mixed in a petri dish. (**b**) class 27 (**c**) class 41 (**d**) class 42. Each class correspond to diatoms species. (**b**–**d**) Phase-contrast map are shown. Diatoms belonging to these three classes have similar morphological features and are used to carry out the tests.

Another dataset, consisting of holographic images of diatoms sampled in natural water bodies, is used for the test. It contains 120 images from 3 classes: 27, 41 and 42. In Figure 5, we show one of the recorded digital holograms of diatoms mixed in a Petri dish (Figure 5a) and three WQPIs from each available class (Figure 5b–d). To the best of our knowledge, this is the first time that deep neural network models devoted to classifying diatoms are trained with an adequate number of simulated phase contrast maps and then tested using holographic images of live samples in a liquid environment. Assessing the accurate classification capability and generalization power of such models would be important because it would show the robustness of the generation method and, on the other hand, would allow the creation of a suitable dataset well-tailored to the application and marine area under test.

### 2.4. Deep Learning Models

Several CNNs architectures have been trained in an end-to-end way and subsequently analyzed in order to establish how each particular architectural choice affects the classification results. In particular, the families of architectures proposed in the recent years that performed better in the ImageNet [39] challenge have been considered in this work and that we can list according to their date of introduction, respectively, in: ResNet [40], DenseNet [41], SENet [42], EfficientNet [43] and RegNet [44]. A fine-tuning procedure has been performed by employing state of the art CNN architectures whose models are provided by authors exploiting an Imagenet dataset for training. In the fine-tuning learning procedure, the weights of the CNN trained on image data provided by Imagenet are reused during training on the Diatoms dataset. In this way, low level features (corners, lines, etc.) learned by the first convolution layers do not need to be learned anymore in the Diatoms domain, but reused by the last convolution layers where actual diatoms features take place. Diatoms images have been provided, during the fine-tuning step, to CNNs input layer as 3 channels’ RGB images, replicating for the second and third layers the same first gray level channel in order to avoid the change of the original CNN topology used in the Imagenet pre-training step.

Firstly, skip layers connection strategies, introduced by ResNet and DenseNet architectures in order to solve the problem of vanishing gradient for very deep architectures, have been investigated. Then, how strategies for modeling inter-dependencies between channels in the convolution layers, introduced with SE-Net by means of the S-E blocks, can improve skip connections-based approaches was studied. Subsequently, strategies for efficiently balancing networks’ depth, width, and resolution introduced in the EfficientNet work, in order to improve performance, have been applied. Finally, instead of focusing on designing individual network instances, a new network design space that parametrizes populations of networks and was recently introduced in [44], has been employed. In particular it makes it possible to combine the advantages of manual design and neural architecture search strategies allowing one to find simple architectures that are easy to understand, build upon, and generalize. In the design space, the basic structure of the network consists of a stem at the input stage followed by a network body for the main part of the calculation, and a final network head for the final classification task. Parameters concerning the stem and head stages are generally kept fixed during parameters’ searching, evaluating only the parameters related to the body part. The latter is composed of 4 stages, each one with progressively reduced resolution. Hence, at each stage it is built up by a sequence of standard identical residual bottleneck blocks with group convolution [45]. The simple and regular networks obtained in the design space are called RegNet and in this work, in particular, the RegNet architecture integrating the Squeeze-and-Excitation (SE) operator as reported in [44] and named RegNetY has been evaluated. For each of the aforementioned CNN strategies, a corresponding family of architectures, each one with increasing complexity, has been evaluated in this work. These have been tested and ranked based on the classification accuracy and computational time required in the training stage. We compared them in the following section.

## 3. Experimental Results

Experiments have been carried out leveraging deep learning architectures as described in the previous section. In order to reduce overfitting problems but still guarantee good generalization properties, training has been performed on a subset of the whole generated dataset. In particular, firstly, a random sampling of 21,000 images from each class, from the whole generated synthetic dataset, has been performed. Secondly, the obtained reduced dataset has been split into training and validation sets sampling randomly 80% and 20% elements, respectively, and preserving labels balancing among classes. Then, a fine-tuning procedure has been performed. The starting models, before fine tuning, have all been pre-trained on the same Imagenet dataset [39]. Each image has been center cropped to a resolution of 224 × 224 pixels and SGD optimizer was employed with learning rate = 0.001, momentum = 0.9 and weight decay = 0.001 parameters. Moreover, an early stopping strategy of five epochs without min loss function improvement on validation set and a maximum number of 200 training epochs have been chosen. At the end of the training procedure, the model that scored the minimum value of loss function was retained and employed in the test phase on the real data. Investigated CNN architectures and related scored results in the test phase are reported in Table 1. All experiments have been performed by means of Pytorch deep learning framework using an NVIDIA Titan RTX GPU card equipped with 24 GB of RAM.

At a first analysis of the results reported in Table 1, it arises that almost all the considered CNN architectures score an accuracy rate greater than 80%, confirming the ability to classify species of diatoms starting from a fully augmented dataset employed for training. Moreover, it is possible to notice that, on the one hand, moving towards deeper implementations generally leads to performance degradation (see for example EfficientNet implementations). On the other hand, less deep architectures score a performance greater than 90% such as SE-ResNet50, EfficientNet-B0 and EfficientNet-B1. This may be due to overfitting problems caused by the greater complexity of the model, in relation to the size of the dataset used for training. Finally, DenseNet121 architecture performance below 80% is related to the difficulty, probably due to the dense block structure employed [36], in extracting highly discriminative features from the simulated images. Table 1 also reports the computational time of the training process for each tested network. In particular, a longer computational time was necessary for EfficientNET-B7, SeNET154 and RegNET6.4GF architecture before convergence, due most probably to the very deep architecture considering the chosen dataset for training. On the other hand, we can see how EfficientNET-B0, EfficientNET-B1 and SE-ResNET50 architectures require the shortest training time.

**Figure 6:**
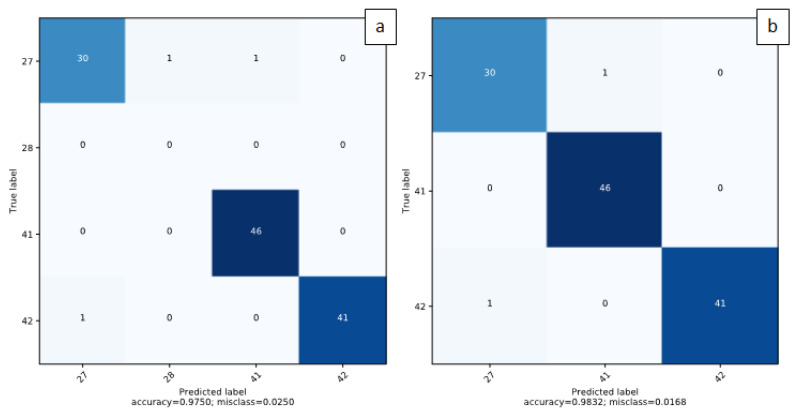
Confusion matrices related to ensemble predictions. (**a**) All output predictions. (**b**) Considering only classes belonging to the test dataset.

In order to further improve the performances achieved by the individual models, a max voting ensemble strategy was used. In particular, models that performed an accuracy greater than 90% have been selected: SE-ResNET50, EfficientNET-B0 and EfficientNET-B1. Obtained results in terms of total accuracy and confusion matrices are reported in the last row of Table 1 and Figure 6, respectively. It can be seen how the use of an ensembling strategy has actually led to an improvement in accuracy, moving from the best result of 95% performed by the SE-ResNET50 architecture to 98% of accuracy obtained with the ensembling confirming the effectiveness of the proposed approach. It is important to note that these three models are the only ones that provide both high accuracy and low computational cost.

Finally, observing the confusion matrix reported in Figure 6a, which samples have been misclassified can be verified. Only three misclassifications occurred. In particular, all samples with label 41 were classified correctly, whereas one only sample with label 42 was misclassified with label 27. Finally, regarding samples with label 27, three misclassifications occurred: one sample classified with label 28 and one sample classified with label 41. The latter was the sole case among the three in which a misclassification occurred with a label not belonging to the test dataset. It should be remembered that the networks have been trained for a prediction of 50 distinct classes, with labels in the range (1,50), while the test dataset consists of samples with labels belonging to a subset of three classes, namely classes with labels 27, 41 and 42. In Figure 6b, we reported the confusion matrix considering only predicted labels belonging to the real dataset.

## 4. Conclusions and Future Works

Mapping the composition of microplankton in seawater is important in the field of environmental monitoring to assess the status of marine habitats [1]. Unicellular algae, namely diatoms, are biomarkers of water quality, since the species populating a certain portion of marine water and the morphometry of each element within a species are affected by the presence of pollutants [2]. Thus, automatic diatoms taxonomy is a highly desirable goal [4,6]. Deep learning approaches require a preliminary stage of training that in turn involves sampling diatoms from seawater and manually labeling each element as belonging to a certain species. This is a very time-consuming process usually made by expert marine biologists. On the other hand, test slides are commercially available where different diatoms species are put in between glass slides while keeping their natural morphology and chloroplast content unaltered. These slides are conventionally used to help taxonomic and research activities, for teaching purposes or as phase-contrast targets to test microscopes and optical systems. In this work, we used one of these slides to generate a wide fully augmented dataset by starting from one generator per class and applying conventional data augmentation techniques. The generator is obtained by acquiring a digital hologram of the test slide and reconstructing the refocused phase-contrast image of each element within the slide. Furthermore, it is remarkable that the training step can be achieved by recording images in a dry environment rather than in a wet environment. The aim of this work was to investigate the capability of ensembles of DCNNs architectures to classify live diatoms in seawater environments after being trained using this set of fully augmented data. We tested thirteen architectures and ranked them on the basis of the classification accuracy achieved in the test stage. To this aim, morphologically similar species have been considered. We selected the three architectures that achieved accuracy higher than 90%, and we implemented a max voting ensemble criterion. In particular, the SE-ResNET50, EfficientNET-B0 and EfficientNET-B1 have been found to overcome the selected accuracy threshold, and the ensemble of these three models reached an accuracy of 98% in distinguishing between the three morphologically similar populations. From one side, this result shows the high generalization power of the DCNNs we considered. Besides, it is apparent how a proper ensemble of different models can boost the classification performance even in this challenging case in which the data used for training are highly correlated.

In this framework, the use of holographic microscopy is pivotal thanks to its flexible focusing capability. Indeed, when diatoms are suspended in a liquid, they occupy different positions in the 3D volume. Thus, for a selected acquisition plane, only few objects are imaged in focus. In conventional optical microscopes, mechanical focus scanning is needed to recover the focus of each object, which can be subject to errors in looking for the best focus condition. Automatic DH refocusing is thus essential to obtain all the objects in sharp focus a posteriori independently of their position, after being captured out-of-focus. In the case of flowing samples, e.g., in imaging flow cytometry for high-throughput environmental monitoring applications, flexible DH refocusing is even more important.

Furthermore, once the hologram is reconstructed, the complex amplitude of the object is available. Thus, accessing such a rich source of information makes the classifier more robust against the introduction of new species and allows the handling of more difficult classification problems. Training networks directly using out of focus images is in principle possible and will be object of further investigations. Besides, the autofocusing process itself could be performed rapidly by using pre-trained networks, thus speeding up the overall processing.

Future works from our group will be devoted to enlarging the set of diatom species captured using proper test slides, and to use field portable holographic microscopes [35,36,37,38] to perform an extensive field sampling campaign to map the distribution of diatoms in specific marine habitats. Within this scope, it is worth pointing out that both inline and off-axis DH imaging schemes could be used [35,36,37,38,46,47,48,49], with their own differences in terms of optical performance and processing required to obtain the phase-contrast maps. Distributing such holographic sensors in larger and larger areas could provide in the near future a taxonomic overview of the micro-plankton classes populating wide marine environments.

## Figures and Tables

**Table 1:** Convolutional Neural Networks (CNNs) accuracy on the test dataset and computational time to train each model.

Model	Accuracy	Computational Time (Minutes)
**EfficientNET-B0**	**0.91**	**414**
**EfficientNET-B1**	**0.94**	**552**
EfficientNET-B2	0.88	588
EfficientNET-B3	0.89	678
EfficientNET-B7	0.72	3198
ResNET50	0.89	455
ResNET101	0.83	664
**SE-ResNET50**	**0.95**	**433**
SE-ResNET101	0.88	744
SeNET154	0.83	5401
DenseNET121	0.73	497
RegNETY6.4GF	0.85	1226
RegNETY4.0GF	0.80	650
		
**ENSEMBLE**	**0.98**	**---**
